# Prostate-Specific Membrane Antigen Expression in Meningioma: A Promising Theranostic Target

**DOI:** 10.1093/jnen/nlac089

**Published:** 2022-09-30

**Authors:** Teddi Tubre, Sean Hacking, Abigail Alexander, Arlen Brickman, Ivana Delalle, Heinrich Elinzano, John E Donahue

**Affiliations:** From the Department of Pathology and Laboratory Medicine, Rhode Island Hospital/Warren Alpert Medical School of Brown University, Providence, Rhode Island, USA; From the Department of Pathology and Laboratory Medicine, Rhode Island Hospital/Warren Alpert Medical School of Brown University, Providence, Rhode Island, USA; From the Department of Pathology and Laboratory Medicine, Rhode Island Hospital/Warren Alpert Medical School of Brown University, Providence, Rhode Island, USA; From the Department of Pathology and Laboratory Medicine, Rhode Island Hospital/Warren Alpert Medical School of Brown University, Providence, Rhode Island, USA; From the Department of Pathology and Laboratory Medicine, Rhode Island Hospital/Warren Alpert Medical School of Brown University, Providence, Rhode Island, USA; Department of Neurology, Rhode Island Hospital/Warren Alpert Medical School of Brown University, Providence, Rhode Island, USA; From the Department of Pathology and Laboratory Medicine, Rhode Island Hospital/Warren Alpert Medical School of Brown University, Providence, Rhode Island, USA

**Keywords:** CD31, Meningioma, Prostate-specific membrane antigen, PSMA, Recurrent, Tumor grade, Tumor marker

## Abstract

Meningioma is the most common intracranial neoplasm, yet there is no effective therapy for recurrent/refractory meningiomas after surgery and radiation. Prostate-specific membrane antigen (PSMA) is an enzyme upregulated on endothelial cells of multiple neoplasms and is being investigated as a theranostic target. Until now, PSMA has not been studied in meningiomas. We aimed to verify PSMA endothelial expression in meningiomas, detect tumor grade variability, and investigate the relationship of PSMA signal with tumor recurrence. We analyzed 96 archival meningiomas including 58 de novo and 38 recurrent specimens. All specimens were stained routinely and immunostained for CD31 and PSMA. Slides were scanned and analyzed producing raw data for images of PSMA, CD31, PSMA/CD31, and PSMA/vasculature. PSMA expression was seen within 98.9% of meningioma samples. In the total cohort, higher-grade tumors had increased expression of raw PSMA and PSMA/CD31, and PSMA/vasculature ratios compared to grade 1 tumors. PSMA expression and PSMA/vasculature ratios (p = 0.0015) were higher in recurrent versus de novo tumors among paired samples. ROC curves demonstrated PSMA/CD31, PSMA/vasculature, and raw CD31 as indicators of tumor recurrence. Thus, PSMA is expressed within endothelial cells of meningiomas, is increased with tumor grade and recurrence, and persists with prior irradiation.

## INTRODUCTION

Meningioma is the most common central nervous system tumor, accounting for 39.0% of all intracranial neoplasms (1). Although the majority of meningiomas are benign (World Health Organization [WHO] grade 1), approximately 10%–15% have a more aggressive course and tend to display rapid tumor growth, recurrence, brain invasion, or rarely, metastatic disease (WHO grades 2 and 3) (2). Currently, surgery and radiation therapy are offered for WHO grade 2 and 3 tumors (3); however, there is no effective therapy for relapsed or refractory meningiomas other than stabilization of disease with systemic therapies. As meningiomas are highly vascularized, there may be a potential role for targeted vascular agents in therapy.

Prostate-specific membrane antigen (PSMA) is a 100-kDa transmembrane peptidase expressed in prostate epithelium that is upregulated in prostate adenocarcinoma and has a positive association with tumor aggressiveness markers such as the Gleason score (4, 5). Additionally, numerous studies have found PSMA to be upregulated in endothelial cells of solid tumors of the breast, lung, thyroid, pancreas, and urothelium, including sarcomas and primary glial tumors where the enzymatic activity may be involved in malignancy-associated angiogenesis (5–10).

Haemels et al (4) in 2020 reported the incidental findings of a relatively low PSMA-avid meningioma via fluorine-18 PSMA (F-PSMA)-1007 positron emission tomography-computed tomography (PET/CT) scan with CT contrast enhancement. Incidental PET/CT PSMA-avid meningiomas are recognized with greater frequency and are documented as known pitfalls of radiotracer analysis (4, 11–13). Thus, it is most likely that endothelial cells of meningiomas express PSMA (4).

As recent studies correlate tumor angiogenesis with PSMA staining and the ability to utilize molecular imaging to monitor PSMA-avid tumors and their response to therapy, targeting PSMA-expressing endothelial cells of brain tumors may prove to be therapeutically useful (14, 15). To our knowledge, PSMA expression in meningioma neovasculature has not been systematically analyzed.

By assessing PSMA endothelial staining in meningiomas grades 1–3, we aimed to demonstrate the variability of PSMA expression in meningiomas in the context of WHO-defined atypical features (including brain invasion, mitoses, presence of necrosis, hypercellularity, small-cell change, macronucleoli, and sheeting architecture), and the Ki-67 proliferation index. Importantly, we evaluated PSMA signal and tumor recurrence in paired patient samples.

## MATERIALS AND METHODS

### Patient Selection and Clinicopathologic Data Acquisition

After institutional board review and approval at Lifespan Hospital with the Warren Alpert Medical School of Brown University, Providence, Rhode Island, formalin-fixed, paraffin-embedded tissues from 69 individual patients who underwent surgical resection between 1996 and 2022 for meningioma, WHO grades 1–3 were obtained. We analyzed 96 archival meningioma cases, selecting 58 de novo (42 grade 1, 15 grade 2, 1 grade 3) and 38 recurrent (16 grade 1, 20 grade 2, and 2 grade 3) specimens. All cases were stained with hematoxylin and eosin (H&E), with subsequent examination by a neuropathologist who selected samples with the most viable tumor to perform immunohistochemical analysis. The selected tissue samples were stained with anti-CD31 to highlight tumor vasculature as a control. Immunohistochemical staining for PSMA was performed to assess PSMA expression within the tumor vasculature.

### Antibodies/Reagents

The primary antibodies used were anti-CD31 Endothelial Cell (concentrate) Clone JC70A purchased from Cell Marque, (Rockland, California) and Prostate-Specific Membrane Antigen (concentrate) Clone 3E6, #M362001-2 purchased from DakoCytomation Inc. (Carpinteria, CA).

### Immunohistochemistry

#### CD31

Four-micrometer-thick sections were cut and baked in an Isotemp oven for 30 minutes at 60°C. Slides were put onto the Dako Link48 for antigen retrieval, and the Flex kit was used for antigen detection. Antigen retrieval was performed for 45 minutes at pH 6 (Citrate). The primary antibody to CD31 was used at a dilution of 1:150 and was automatically titrated onto the slides and subsequently incubated for 30 minutes. The universal secondary antibody was automatically dispensed and incubated for 20 minutes. Slides were removed from the Link48 and put into deionized water mixture, counterstained with hematoxylin, 0.2% ammonia water for 1 minute, and then run under deionized water for 5 minutes. Slides were then dehydrated and coverslipped.

#### PSMA Primary Antibody

Four-micrometer-thick sections were cut and baked in an Isotemp oven for 30 minutes at 60°C. Slides were put onto the Dako Link48 for antigen retrieval, and the Flex kit with mouse linker was used for antigen detection. Antigen retrieval was performed for 45 minutes at pH 9 (EDTA). The primary antibody for PSMA was used at a dilution of 1:10 and was automatically titrated onto the slides and subsequently incubated for 10 minutes. The universal secondary antibody was automatically dispensed and incubated for 15 minutes. Slides were removed from the Link48 and put into a deionized water mixture, counterstained with hematoxylin, 0.2% ammonia water for 1 minute, and then run under deionized water for 5 minutes. Slides were then dehydrated and coverslipped.

### Slide Scanning

Both H&E and immunostained sections were scanned on a Leica Aperio CS2 WSI slide scanner at 20× magnification objective lens (0.5 μm/pixel). All of the digital slides were then downloaded from the Aperio vendor agnostic whole slide image (WSI) viewer in the standard visualization system image format with variable degrees of JPEG image compression. WSIs were deidentified and labeled with a sticker and numbered 1–96. Data transfer and storage were undertaken in a Health Insurance Portability and Accountability Act of 1996 (HIPPA)-compliant manner.

### Machine Learning

QuPath (version 0.2) (16) was utilized as an open access solution to machine learning for WSIs. All WSI files were imported and orientated appropriately. A supervised Random Forest machine learning model was developed and trained from annotated patches selected from a randomized training patient cohort. An average of 5 patches was selected from each WSI within the training set, and a total of 200 training patches were utilized for model development. The training set was selected to represent tissue diversity, including tumor histologic subtypes, grade, and other pathologic factors such as mitoses and atypia. The demographics of the training cohort can be found in the Supplementary Material.

Vascular density was calculated from H&E-stained slides, while PSMA and CD31 were calculated from IHC-stained slides. Ground truth for vasculature as defined on H&E-stained slides included 3 shared layers of the vessel wall for arteries and veins (tunica intima, media, and externa), with small arteries and arterioles usually containing 1 or 2 layers of smooth muscle cells, and often absence of the external elastic membrane in arterioles. Additionally, continuous capillaries are present in the central nervous system (17), ranging from 4 to 10 microns with a reduced vessel wall comprised endothelium surrounded by connective tissue. On H&E, a basement membrane that houses pericytes can also be visible. For PSMA and CD31, positive ground truth was defined as cytoplasmic and/or membranous unequivocal vascular staining of intensity above background. Negative staining was defined as the absence of any detectable IHC staining, characterized by a pale gray discoloration.

### Assessment of Biomarker Expression

**FIGURE 1.**
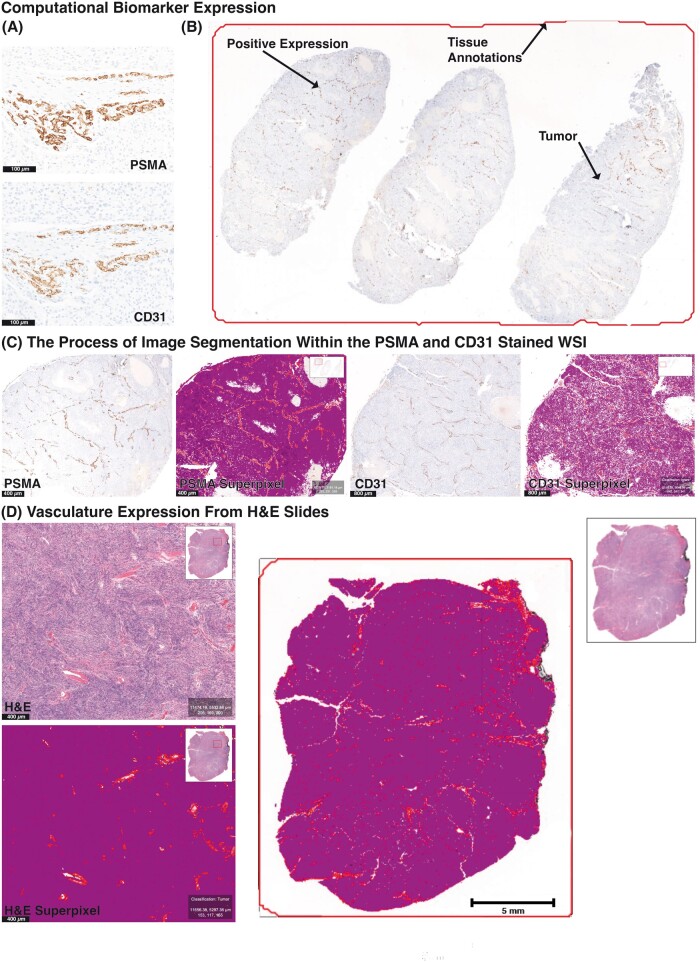
Computational biomarker expression quantified by machine learning-based classification. **(A)** Hotspots demonstrating PSMA and CD31 immunohistochemical expression within meningioma endothelial cells, **(B)** annotated WSI with positive PSMA expression within tumor endothelium and adjacent negative tumor cells, **(C)** the process of image segmentation within the PSMA and CD31 stained WSI utilizing grouped pixels with common characteristics (superpixels) of the respective IHC, and **(D)** vasculature expression from H&E-stained slides and the corresponding superpixel. The end result differentiates PSMA and CD31 colocalization within meningioma endothelial cells, involved vascular walls, and tumor cells without expression of either PSMA or CD31.

An overview biomarker quantification from WSIs is shown in Figure 1. Briefly, the models were developed under very high (downsample = 2.00) resolution. Features were set as: “Default multiscale features.” The live prediction parameter was set in default as: “Number of threads: −1. Maximum samples: 100 000. RNG seed: 100.” Preprocessing parameters were set to default as follows: “Feature normalization: None. Feature reduction: No feature reduction. PCA retained variance: 0. Boundary strategy: Skip. Boundary thickness: 0 pixels.” The final classifier grouped superpixels automatically groups based on pixel similarities between different labeled cellular populations. This was demonstrated as red (CD31- and PSMA-positive endothelial cells), yellow (negative endothelial cells), and purple (tumor). Percentages were calculated for each of the populations and features including raw CD31, raw PSMA, PSMA/CD31 ratio of coexpression, and a PSMA/vasculature ratio were determined.

### Statistical Analysis

The dependent variables (raw CD31, raw PSMA, PSMA/CD31 ratio, and PSMA/vasculature ratio) were examined in International Business Machines Corporation Statistical Package for the Social Sciences (IBM SPSS) 27 as a function of dichotomized categorical values (numerical scale) seen in Tables 1 and 2. Medians and their significance were compared with a Mann-Whitney U-test. Receiver operator curves (ROCs) were calculated graphically to demonstrate the connection and trade-off between clinical sensitivity and specificity for all possible cut-off points for the computational biomarkers in question. All tests were 2-sided, with p values <0.05 considered statistically significant.

**TABLE 1.** Computational Biomarkers in Relation to Clinical/Pathologic Features and Site

Biomarker	CD31	p	PSMA	p	PSMA/CD31	p	PSMA/V	p
Age (96)		0.263		0.010		0.010		0.002
<50 (23)	2.81		0.176		0.0855		0.0133	
>50 (73)	1.66		0.972		0.373		0.0378	
Sex (96)		0.222		0.442		0.607		0.418
Male (36)	2.67		0.741		0.391		0.0267	
Female (60)	1.59		0.596		0.202		0.0206	
Procedure (96)		0.876		0.135		0.262		0.217
Craniotomy (82)	1.71		0.557		0.236		0.206	
Other (14)	1.99		1.83		0.953		0.0511	
Size (96)		0.006		0.793		0.038		0.909
<4 cm (55)	0.104		0.630		0.400		0.0225	
≥4 cm (41)	2.67		0.603		0.162		0.0218	
Grade (96)		0.584		0.039		0.034		0.015
Grade 1 (58)	1.93		0.483		0.219		0.0164	
Grade 2, 3 (38)	1.71		0.810		0.442		0.0361	
Ki-67 (85)		0.892		0.118		0.121		0.085
<4% (33)	1.64		0.314		0.139		0.0148	
≥4% (52)	1.94		0.697		0.319		0.0298	
Hypercellularity (73)		0.828		0.221		0.0165		0.362
Present (32)	1.65		0.715		0.442		0.0234	
Absent (41)	1.94		0.386		0.201		0.0156	
Sheeting (59)		0.124		0.508		0.711		0.287
Present (11)	3.06		0.386		0.201		0.0344	
Absent (48)	1.62		0.463		0.161		0.0179	
Small cell change (60)		0.827		0.733		0.916		1.000
Present (14)	1.68		0.310		0.167		0.0150	
Absent (46)	1.94		0.581		0.208		0.0252	
Macronucleoli (62)		0.690		0.226		0.305		0.226
Present (25)	1.66		0.559		0.278		0.0368	
Absent (38)	2.04		0.547		0.161		0.0213	
Necrosis (82)		0.963		0.304		0.197		0.274
Present (36)	1.91		0.624		0.372		0.0435	
Absent (45)	1.94		0.575		0.208		0.0222	
Mitoses (89)		0.199		0.157		0.024		0.021
<4 mitoses/10 HPF (66)	1.93		0.557		0.219		0.0200	
≥4 mitoses/10 HFP (23)	1.29		0.972		1.65		0.0500	
Brain invasion (71)		0.639		0.436	0.292	0.332		0.668
Present (11)	1.66		0.840		0.19		0.0225	
Absent (60)	1.93		0.463				0.0210	
Psammoma bodies (64)		0.605		0.172		0.354		0.066
Present (36)	1.60		0.529		0.187		0.0209	
Absent (28)	2.66		0.801		0.283		0.0588	
Radiation (91)		0.228		0.369		0.095		0.390
Yes (19)	1.58		0.73		0.868		0.0214	
No (72)	1.92		0.557		0.224		0.0213	
Site (73)		0.714		0.286		0.191		0.739
Convexity (57)	2.61		0.630		0.214		0.0253	
Skull base (16)	1.61		0.885		0.684		0.0211	

**TABLE 2.** Computational Biomarkers in Relation to Clinical and Pathologic Features in De Novo Tumors

Biomarker	CD31	p	PSMA	p	PSMA/CD31	p	PSMA/V	p
Age (58)		0.323		0.20		0.005		0.013
<50 (12)	3.56		0.121		0.0290		0.00526	
>50 (46)	1.90		0.600		0.219		0.0238	
Sex (58)		0.014		0.768		0.442		0.545
Male (20)	3.53		0.492		0.162		0.0220	
Female (38)	1.59		0.411		0.186		0.0184	
Procedure (58)		0.537		0.497		0.241		0.484
Craniotomy (48)	2.21		0.409		0.150		0.0185	
Other (10)	1.89		1.01		0.391		0.0292	
Prior radiation (58)		0.921		0.260		0.360		0.087
Yes (3)	1.92		0.603		0.289		0.111	
No (55)	1.94		0.417		0.184		0.0173	
Size (58)		0.125		0.744		0.090		0.950
<4 cm (30)	1.61		0.493		0.236		0.0200	
≥4 cm (28)	2.74		0.411		0.106		0.0200	
Grade (58)		0.754		0.821		0.958		0.651
Grade 1 (42)	2.23		0.434		0.186		0.0160	
Grade 2, 3 (16)	1.81		0.495		0.182		0.0217	
Ki-67 (51)		0.650		0.850		0.835		0.880
<4% (23)	2.91		0.436		0.138		0.0203	
≥4% (28)	2.59		0.330		0.133		0.0203	
Hypercellularity (49)		0.555		0.349		0.238		0.583
Present (29)	1.65		0.608		0.240		0.0228	
Absent (20)	2.91		0.257		0.110		0.0133	
Sheeting (41)		0.176		0.322		0.711		0.487
Present (8)	4.23		0.583		0.194		0.0407	
Absent (33)	2.51		0.257		0.103		0.0132	
Small cell change (41)		1.00		0.601		0.777		0.777
Present (10)	2.23		0.185		0.0872		0.00912	
Absent (31)	2.91		0.559		0.138		0.0208	
Macronucleoli (44)		0.379		0.448		0.656		0.443
Present (17)	3.50		0.436		0.132		0.0148	
Absent (27)	2.86		0.411		0.108		0.0206	
Necrosis (51)		0.469		0.512		0.500		0.512
Present (20)	2.74		0.493		0.213		0.0361	
Absent (31)	2.67		0.436		0.138		0.0148	
Mitoses (57)		0.531		0.876		0.639		0.557
<4 mitoses/10 HPF (45)	2.51		0.436		0.205		0.0200	
≥4 mitoses/10 HPF (12)	1.47		0.517		0.205		0.0350	
Brain invasion (48)		0.773		0.637		0.727		0.773
Present (6)	1.63		0.0821		0.174		0.0217	
Absent (42)	2.66		0.411		0.161		0.0175	
Psammoma bodies (48)		0.788		0.703		0.543		0.439
Present (25)	1.94		0.559		0.138		0.0209	
Absent (23)	2.81		0.436		0.214		0.0203	

## RESULTS

### Patient Cohort

In total, 96 specimens (both de novo tumors and recurrent tumors) were included on the WSI open access solution to machine learning. These were derived from de novo (primary tumors) and recurrent (first, second, and third recurrences) from 69 patients, of which 37 cases comprised paired patient samples (both de novo and recurrent specimens). A total of 58 de novo (42 grade 1, 15 grade 2, and 1 grade 3) specimens and 38 recurrent (16 grade 1, 20 grade 2, and 2 grade 3) specimens were evaluated and are represented in Table 1. The mean age of the patient cohort was 58 years, of which 23 patients were under the age of 50 years, and 73 patients were 50 years of age or older. A total of 60 patients were female, and 36 were male. While 82 patients underwent craniotomy for resection of tumor, 14 patients underwent other procedures, including excision or biopsy. Neuropathologic reports indicated 55 patients had a tumor that was <4 cm and 41 patients had a tumor that was ≥4 cm in greatest dimension on gross examination. A total of 58 specimens were grade 1 tumors, and 38 specimens were grades 2–3. Of note, 12/22 (54.5%) of patients in the paired group had received radiotherapy. While higher-grade tumors revealed the presence or absence of necrosis, brain invasion, hypercellularity, sheeting architecture, macronucleoli, small-cell change, and elevated Ki-67 index of proliferation, there were no significant differences within this group either in the de novo specimens or entire patient cohort in any of these defining features. Staining for Ki-67, a marker of cellular proliferation, was estimated or quantified at <4% in 33 specimens, and ≥4% in 52 specimens. Hypercellularity was reported present in 32 specimens and absent in 41 specimens. Sheeting architecture was reported present in 11 specimens and absent in 48 specimens. Small-cell change was reported present in 14 specimens and absent in 46 specimens. The presence of macronucleoli was reported in 25 specimens and absent in 38 specimens. The presence of spontaneous necrosis was reported present in 36 specimens and absent in 45 specimens. Brain invasion was present in 11 specimens and absent in 60 specimens. Psammoma bodies were reported present in 35 specimens and absent in 28 specimens. Notably, specimens with <4 mitoses per 10 high-power fields were seen in 66 cases and ≥4 mitoses per 10 high-power fields in 23 cases.

### Biomarker Expression in Total Cohort

**FIGURE 2.**
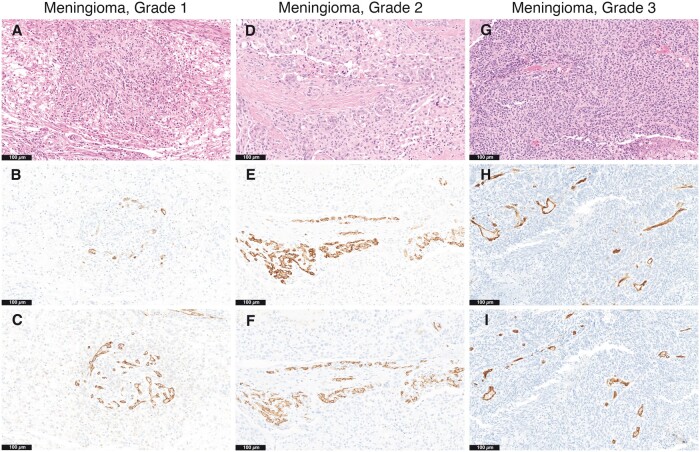
Prostate-specific membrane antigen (PSMA) and CD31 colocalization across grades in total cohort. Hematoxylin and eosin (H&E)-stained section of CNS WHO Grade 1 meningioma, transitional subtype **(A)** with PSMA **(B)** and CD31 **(C)** immunostains in tumor vasculature; raw PSMA expression (0.16), raw CD31 expression (7.07), and PSMA/CD31 ratio (0.02). H&E-stained section of CNS WHO Grade 2 meningioma, transitional subtype **(D)** with PSMA **(E)** and CD31 **(F)** immunostains in prominent vessels; raw PSMA expression (7.85), raw CD31 expression (4.15), and PSMA/CD31 ratio (1.89). H&E-stained section of CNS WHO Grade 3 meningioma of mixed architectural features **(G)** with PSMA **(H)** and CD31 **(I)**; raw PSMA (5.52), raw CD31 (1.29), and PSMA/CD31 ratio (4.26). Across the total cohort, raw PSMA, raw C31, and the PSMA/CD31 ratio increased with higher grade tumors and recurrence. Original magnifications: A–I = 200×.

The entire cohort consists of 96 patient samples. Overall, patients ≥50 years old had increased raw PSMA (p = 0.010), PSMA/CD31 ratio (p = 0.010), and PSMA/vasculature ratio (p = 0.002) compared with patients <50 years of age. Patients with tumor size ≥4 cm had increased raw CD31 (p = 0.006) and an increased PSMA/CD31 ratio (p = 0.038) compared to patients with tumor sizes <4 cm in greatest dimension grossly. Higher-grade tumors (grades 2–3) had increased expression of raw PSMA (p = 0.039), PSMA/CD31 ratio (p = 0.034), and PSMA/vasculature ratio (p = 0.015) compared to grade 1 tumors. Biomarker expression among the total cohort with regard to increased raw PSMA and PSMA/CD31 ratios is shown in Figure 2. Additionally, patients with ≥4 mitoses per 10 high-power fields had increased PSMA/CD31 ratio (p = 0.024) and PSMA/vasculature ratio (p = 0.021) compared to patient samples with <4 mitoses per 10 high-power fields.

### Biomarker Expression in De Novo Tumors

Patients with de novo grade 1 meningiomas had no significant differences in raw PSMA expression (p = 0.821), raw CD31 (p = 0.754), PSMA/CD31 ratio (p = 0.958), or PSMA/vasculature ratio (p = 0.651) compared to de novo grade 2–3 tumors represented in Table 2. Of the de novo cohort, patient age ≥50 years old correlated with higher PSMA/CD31 ratio (p = 0.005) and PSMA/vasculature ratio (p = 0.013) compared to patients <50 years of age. Males had a higher raw CD31 expression (p = 0.014) compared to females.

### Biomarker Expression in Relation to Localization

The tumors in this cohort localize as follows: lobar (frontal, parietal, temporal, occipital), intraventricular, sphenoid, intraorbital/optic nerve, parasagittal, midline, posterior fossa, temporal fossa, frontal skull base, spine, and unspecified. According to the 2021 WHO brain tumor classification scheme (18), convexity and the majority of spinal meningiomas have similar molecular findings (loss of chromosome 22q and/or *NF2* mutations), while skull base meningiomas demonstrate different mutations (*AKT1, TRAF7, SMO, and PIK3CA*). Accordingly, we analyzed 2 groups: one combining lobar, parasagittal, and spine into one group (57 cases), and sphenoid, optic nerve/intraorbital, posterior fossa, temporal fossa, and frontal skull base together in a second (“skull base”) group (16 cases). (“midline,” “intraventricular,” and “unspecified” were omitted from this analysis.) There were no significant differences in raw PSMA expression (p = 0.286), raw CD31 expression (p = 0.714), PSMA/CD31 ratio (p = 0.191), and PSMA/vasculature ratio (p = 0.739).

### Biomarker Expression in Relation to Recurrence

Based on initial tumor biomarker expression, the following ROC curves were found: Raw CD31 had an AUC of 0.7278 with a SE of 0.05965 and 95% confidence interval between 0.6108 to 0.8447 (p < 0.001). Raw PSMA had an AUC of 0.5642 with a SE of 0.0734 and a 95% confidence interval of 04202 to 0.7082 (p = 0.394). PSMA/CD31 AUC of 0.7843 with a SE of 0.05651 and a 95% confidence interval of 0.6736 to 0.8951 (p < 0.001). PSMA/vasculature had an AUC of 0.6456 with a SE of 0.06483 with a 95% confidence interval of 0.5185 to 0.7726 (p = 0.033).

**FIGURE 3.**
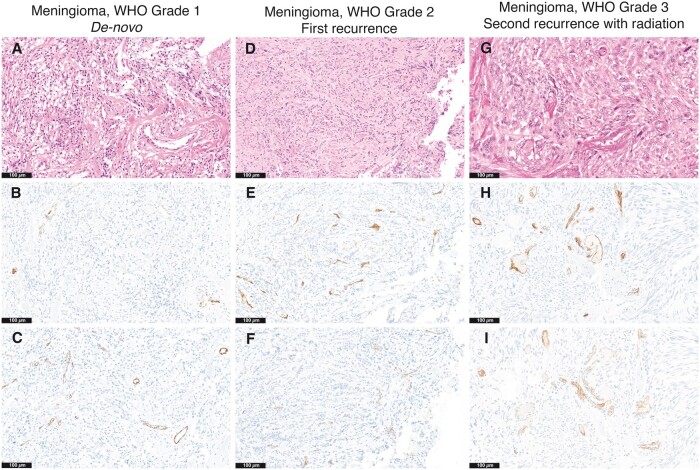
Prostate-specific membrane antigen (PSMA) and CD31 coexpression across paired patient sample. Hematoxylin and eosin (H&E)-stained section of the patient’s de novo CNS WHO Grade 1 meningioma of meningothelial subtype **(A)** with PSMA **(B)** and CD31 **(C)** colocalization within tumor endothelial cells with raw PSMA (2.22), raw CD31 (2.13), and PSMA/CD31 ratio (1.04). Approximately 7 years later, the tumor recurred, H&E-stained section of CNS WHO Grade 2 meningioma of meningothelial subtype **(D)** with PSMA **(E)**, and CD31 **(F)** coexpression in tumor neovasculature with raw PSMA (0.65), raw CD31 (0.39), and PSMA/CD31 ratio (1.65). The patient underwent gross total resection with subsequent radiation; however, approximately 8 years later, the tumor recurred. The H&E-stained section of CNS WHO grade 2 meningioma of predominantly transitional subtype with radiation-induced changes **(G)**, PSMA **(H)** and CD31 **(I)** retained coexpression within tumor neovasculature with raw PSMA (0.75), raw CD31 (0.44), and PSMA/CD31 ratio (1.7). In this case, the PSMA/CD31 ratio increased with recurrence and grade and was retained even after radiation treatment. Original magnifications: A–I = 200×.

**FIGURE 4.**
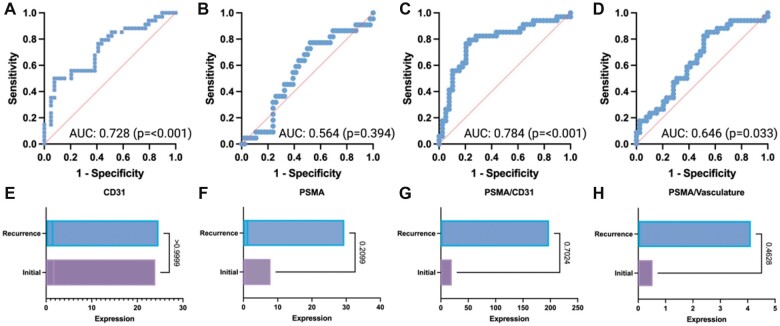
Computational biomarkers in relation to recurrence at a patient level with Wilcoxon signed rank test for paired samples. **(A)** AUC for CD31, **(B)** AUC for PSMA, **(C)** AUC for PSMA/CD31 ratio, **(D)** AUC for PSMA/vasculature ratio, **(E)** Wilcoxon signed rank test for CD31 between paired samples, **(F)** Wilcoxon signed rank test for PSMA between paired samples, **(G)** Wilcoxon signed rank test for PSMA/CD31 ratio between paired samples, and **(H)** Wilcoxon signed rank test for PSMA/vasculature ratio between paired samples. Across paired patient samples, the raw CD31 expression, PSMA/CD31 ratio, and PSMA/vasculature ratio were indicators of tumor recurrence.

The median raw CD31 expression was 1.675 in the initial and 1.40 in the paired recurrence specimens with a median difference of 0.1500 (p = 0.999). The median raw PSMA expression was 0.66 in the initial and 1.17 in the paired recurrence specimens with a median difference of 0.2215 (p = 0.2099). The median PSMA/CD31 ratio expression was 0.734 in the initial and 1.048 in the paired recurrence specimens with a median difference of 0.03019 (p = 0.7024). The raw PSMA and CD31 colocalization in an individual paired patient sample is shown in Figure 3. The median PSMA/vasculature ratio expression was 0.0387 in the initial and 0.0389 in the paired recurrence specimens with a median difference of 0.004605 (p = 0.4628). AUC for biomarkers and the Wilcoxon matched pairs signed rank test are shown in Figure 4.

## DISCUSSION

The present study measured the expression of PSMA in meningiomas to explore the feasibility of targeting PSMA in initial diagnostic, subsequent PET/CT monitoring, and in the selective delivery of PSMA-targeted therapeutic agents. We found that 98.9% of meningioma specimens expressed PSMA within their endothelial cells. Additionally, across the total cohort PSMA expression was higher in tumors grades 2–3 when compared to grade 1 and in recurrent tumors compared to de novo tumors. As the vast majority of meningiomas are benign, the mainstay of therapy for grade 1 tumors is gross total resection. In tumors grades 2–3, patients are additionally treated with radiation therapy subsequent to surgical resection. Unfortunately, higher-grade tumors have increased aggressiveness, rates of recurrence, and refractoriness to therapy, including the presence of atypical features and a Ki-67 index of proliferation ≥4% (19). In tumors that recur, the standard of care relies on stabilization of disease by systemic therapies as there are no currently documented effective therapies for relapsed or recurrent tumors. Therefore, once confirming PSMA expression within the neovasculature of meningiomas, we sought to identify the variability of expression of PSMA within the neovasculature of recurrent tumors in paired patient samples. Our findings suggest that PSMA expression is not only limited to the tumor endothelial cells but extends within the vascular walls as highlighted by PSMA/vasculature ratios in recurrent patient cases, and that PSMA expression remains upregulated in recurrent tumors that have had prior radiation therapy.

Diagnostic imaging has made great changes to the early diagnosis and management of patients with PSMA-expressing tumors, namely those with a history of prostatic adenocarcinoma. 68GA-PSMA-11 was one the first positron emitting radionucleotides to be used in diagnostic imaging (15). Currently, the utility of 68GA PSMA-11 lies in the ability to localize within tumors that are somatostatin receptor (SSTR2)-positive (15). Thus, SSTR2-positive tumors, through labeling with PSMA ligands, can be targeted, resulting in an increased uptake of 68GA PSMA-11 (15). As the majority of meningiomas express SSTR2, it is expected that meningiomas also have uptake by 68GA PSMA-11. Galldiks et al (20) showed a correlation between 68GA-DOTATATE-uptake and WHO grade in meningiomas but a major limitation to monitoring was a lack of tumor specificity and complete loss in anaplastic meningiomas. Additional studies by Sasikumar et al (15) concluded that 68GA PSMA-11 can be thought of as a potentially useful imaging tool in the evaluation of brain lesions, as radiotracers targeting the enzyme PSMA could potentially target the neovasculature of brain tumors. These findings were confirmed by multiple studies that reported expression of PSMA in many solid tumors, primary gliomas, and brain metastases (6–10). In addition, case reports are becoming more frequent in which maintenance imaging of patients with a known history of prostate cancer with 68GA PSMA-11 has led to the incidental finding of PSMA-avid meningiomas (4, 21).

Numerous studies have shown that PSMA has not been reported in normal vasculature and is the only specific neovascular target currently known (22). Additionally, PSMA has been reportedly expressed by tumor endothelium in nearly all solid tumor types without expression in adjacent tumor cells or the normal vasculature (22–24). Multiple phase I clinical trials utilizing combinations of antibody with J591, which targets the extracellular domain of PSMA, have revealed selective targeting, good tumor localization, and tumor toxicity (22–24). These results suggest that PSMA may be a key regulatory agent of angiogenic activity, as preclinical data has demonstrated severely impaired angiogenesis in PSMA-null animals (23).

Our findings confirm that PSMA is expressed within the neovasculature of meningiomas. While de novo meningiomas had no significant expression of raw PSMA when comparing de novo grade 1 versus grades 2–3 tumors, the raw PSMA expression relative to grade was increased in recurrent tumors. This finding suggests that the angiogenesis of tumor neovascularization may increase with recurrence and may function as a driver of increased angiogenesis. As PSMA expression is increased in recurrent tumors, irrespective of prior radiation therapy, it is likely that PSMA is a driver of the neovascularization rather than as a tumor response to radiation therapy. Our findings show that in tumors that are recurrent, of higher grade, and have undergone radiation therapy, PSMA expression remains intact, and the raw expression is increased. Thus, as PSMA is unregulated in patients with recurrent tumor, the neovasculature is potentially targetable by PSMA directed therapies.

Our observed variability of PSMA staining between grade 1 tumors of differing subtypes needs to be more comprehensively studied. Of the de novo grade 1 tumors investigated, there were extremes of PSMA staining in relation to the variability of tumor neovasculature; namely, angiomatous samples were at the higher end of raw PSMA expression, while fibrous subtypes were at the lower end of the spectrum. Additionally, heterogeneity of tumor staining in the tissue sample should be studied more comprehensively. As the majority of de novo grades 1–3 tumors were acquired before the patients underwent radiation therapy, the intratumoral heterogeneity is most likely due to a combination of the proliferation of angiogenic vessels per subtype, and in cases of recurrence, to grade. As identified in prostate cancer, expression of PSMA is highest in higher-grade tumors (7).

The cutoff for patient age of 50 was instituted as the mean age of our entire cohort was 58 years old. Other variables, including the cutoff of <4 and ≥4 for both mitoses per 10 high-power fields and the rate of proliferation as determined by estimation or quantification of immunolabeling with Ki-67, were based on the 2021 CNS WHO criteria for evaluation of Ki-67 as a marker of tumor aggressiveness and recurrence and the number of mitoses as diagnostic criteria involved in the grading of meningiomas (18). Additional histological criteria, including brain invasion and the presence or absence of atypical features, were also evaluated as they are known grading criteria (18).

Our data follow the studies of expression of PSMA in primary glial tumors (6, 7). Wernicke et al (6) and Nomura et al (7) reported PSMA immunoreactivity in the neovasculature of all glioblastoma specimens tested, although they discuss that an earlier study had found that glioblastoma specimens were negative for PSMA expression. The disparate results were thought to be secondary to the quality of the tumor tissue and ascribed to minimal stroma in the tested samples and to the use of different anti-PSMA antibodies. Therefore, we chose to utilize the same 3E6 mAb used by Wernicke et al and Nomura et al. The single case in our study that did not express PSMA was a de novo tumor with poor preservation for immunohistochemical analysis.

Our study has a few limitations. Although studies have correlated mitotic index with the mitosis marker phosphohistone H3 (pHH3) (25, 26), we do not utilize pHH3 immunohistochemistry at our institution. We use the Ki-67 labeling index. Neither pHH3 nor Ki-67 IHC are official WHO histologic criteria for grading meningiomas (18). A recent meta-analysis has shown a good correlation between Ki-67 labeling index and prognosis (27). Molecular testing is also not part of our routine workup of meningiomas. Molecular analysis is becoming increasingly important in meningioma diagnostics, particularly for higher-grade tumors (18). Correlating PSMA expression with pHH3 immunohistochemistry and molecular testing in meningiomas are avenues for future research.

Machine learning was utilized in this study to completely objectify the results and eliminate human variance in light microscopic interpretation. The goal is to perform reproducible scoring of PSMA in meningioma along with other biomarkers from WSI. However, further research and validation of computational biomarkers is required before coming up with such a system for routine clinical pathologic use. One such project would be to correlate molecular imaging for PSMA with tissue expression to see if the PSMA positivity has relevance to the patient, particularly regarding anti-PSMA therapy. Determining cutoffs for PSMA expression could be coupled with more advanced machine learning and multi-omics approaches to predict treatment response to PSMA radioligand therapy.

PSMA radioligand therapy has emerged as a significant therapeutic in prostate cancer (28), where small-molecule PSMA inhibitors are labeled with both beta- and alpha-emitting radioisotopes, with different energy levels and path lengths. Beta-emitting radioisotopes include lutetium-177 (^177^Lu), favored for use in prostate cancer secondary to minimal tissue penetration and a long half-life, which facilitates a higher degree of radiation. Radioligand therapy with ^177^Lu-PSMA-617 has been shown to prolong imaging-based survival when used in conjunction with standard of care for patients with PSMA-positive, advanced metastatic castration-resistant prostate cancer (29).

Lastly, since the current study focused mainly on banked tumor tissue, we did not look into the relationship between PSMA expression in meningioma and neuroimaging features such as peritumoral edema. Quantitative and qualitative information derived from perfusion MRI analyzing peritumoral edema may suggest the histological grade of meningioma preoperatively and potentially predict likely patient outcomes (30). This important correlation between imaging features and histopathological characteristics in meningioma will be studied in future clinical trials incorporating advanced neuroimaging techniques using PET/MRI hybrid and radiomics.

In conclusion, the present study suggests that PSMA expression in endothelial cells of meningiomas increases with recurrence and grade. These results provide a rationale for meningioma treatment in accordance with PSMA expression and call for clinical trial testing utilizing PSMA-targeted therapies as alternative diagnostic, therapeutic, and maintenance approaches for recurrent/refractory meningiomas.

## Supplementary Material

nlac089_Supplementary_Data

## Notes

Disclosure: Preliminary versions of this work were presented as posters at annual meetings of the American Association of Neuropathologists and American Society of Clinical Oncology.
